# Supplementary material for: Multi‐dataset identification of innovative feature genes and molecular mechanisms in keratoconus
Source: J Cell Mol Med. 2024 Sep 19;28(18):e70079. doi: 10.1111/jcmm.70079 (PMC11412914; doi:10.1111/jcmm.70079)
Supplement: Supplementary file 2 — Material S2. [file JCMM-28-e70079-s002.zip › 7.GSEA analysis/1.ARL11/KEGG_GLYOXYLATE_AND_DICARBOXYLATE_METABOLISM.html]

Details for gene set KEGG\_GLYOXYLATE\_AND\_DICARBOXYLATE\_METABOLISM[GSEA]

|  || Dataset | ARL11.group.cls#h\_versus\_l |
| Phenotype | group.cls#h\_versus\_l |
| Upregulated in class | h |
| GeneSet | KEGG\_GLYOXYLATE\_AND\_DICARBOXYLATE\_METABOLISM |
| Enrichment Score (ES) | 0.40775278 |
| Normalized Enrichment Score (NES) | 1.1699991 |
| Nominal p-value | 0.2917505 |
| FDR q-value | 1.0 |
| FWER p-Value | 0.993 |
Table: GSEA Results Summary

  

Fig 1: Enrichment plot: KEGG\_GLYOXYLATE\_AND\_DICARBOXYLATE\_METABOLISM      
 Profile of the Running ES Score & Positions of GeneSet Members on the Rank Ordered List

  

| SYMBOL | TITLE | RANK IN GENE LIST | RANK METRIC SCORE | RUNNING ES | CORE ENRICHMENT || 1 | HAO1 | na | 1788 | 0.312 | 0.2619 | Yes |
| 2 | GLYCTK | na | 3983 | 0.201 | 0.4009 | Yes |
| 3 | HAO2 | na | 7381 | 0.104 | 0.4078 | Yes |
| 4 | PGP | na | 14216 | 0.017 | 0.2282 | No |
| 5 | AFMID | na | 14714 | 0.013 | 0.2274 | No |
| 6 | ACO2 | na | 15444 | 0.008 | 0.2144 | No |
| 7 | CS | na | 15543 | 0.007 | 0.2188 | No |
| 8 | MDH2 | na | 16386 | 0.001 | 0.1956 | No |
| 9 | GRHPR | na | 18147 | -0.011 | 0.1564 | No |
| 10 | MDH1 | na | 18516 | -0.014 | 0.1596 | No |
| 11 | MTHFD2L | na | 18735 | -0.015 | 0.1686 | No |
| 12 | MTHFD1L | na | 19886 | -0.023 | 0.1586 | No |
| 13 | MTHFD1 | na | 21043 | -0.032 | 0.1571 | No |
| 14 | MTHFD2 | na | 24721 | -0.072 | 0.1239 | No |
| 15 | ACO1 | na | 25363 | -0.081 | 0.1868 | No |
| 16 | HYI | na | 25480 | -0.083 | 0.2664 | No |
Table: GSEA details [plain text format]

  

Fig 2: KEGG\_GLYOXYLATE\_AND\_DICARBOXYLATE\_METABOLISM      
 Blue-Pink O' Gram in the Space of the Analyzed GeneSet

  

Fig 3: KEGG\_GLYOXYLATE\_AND\_DICARBOXYLATE\_METABOLISM: Random ES distribution      
 Gene set null distribution of ES for **KEGG\_GLYOXYLATE\_AND\_DICARBOXYLATE\_METABOLISM**
